# Emerging pathological mechanisms of Alzheimer’s disease pathogenesis: from neuroimmune interactions to intercellular communication

**DOI:** 10.3389/fnagi.2026.1748418

**Published:** 2026-03-04

**Authors:** Rutong Wang, Yingqi Feng, Ziyu Zhou, Jiajun Jiang, Runze Zhang, Wenhui Zou, Haotian Yang, Wenbo Lv, Shen Yang

**Affiliations:** 1 Department of Neurology, The First People’s Hospital of Xiangtan, Xiangtan, Hunan, China; 2 Hengyang Medical School, Clinical Anatomy and Reproductive Medicine Application Institute, University of South China, Hengyang, Hunan, China

**Keywords:** Alzheimer’s disease (AD), complement system, extracellular vesicles, microglia, neuroimmunology

## Abstract

Alzheimer’s disease (AD) research has transcended the traditional paradigm centered on amyloid-beta (Aβ) shifting toward a neuroimmune network perspective. This article systematically elucidates the evolving mechanisms underlying disease progression, from neuroimmune interactions to intercellular communication. Studies indicate that microglial and astrocytic dysfunctions are key contributors to disease progression, operating within a complex multifactorial framework. Upon transformation into disease-associated microglia (DAM), microglia exhibit a significant decline in Aβ clearance capacity and release a plethora of pro-inflammatory factors, exacerbating neuroinflammation and neuronal damage. Concurrently, astrocytes lose their homeostatic support functions and acquire neurotoxic properties. Intercellular communication molecules play pivotal roles as key mediators. The cytokine/chemokine network sustains a chronic inflammatory milieu; extracellular vesicles (EVs) facilitate the propagation of Aβ and tau pathologies; and the complement system (e.g., C1q) transitions from physiological synaptic pruning to pathological synaptic engulfment. Furthermore, peripheral immune cell infiltration and gut-brain axis dysregulation further expand the pathological scope. Consequently, therapeutic strategies are evolving towards multi-target interventions, including precise immune modulation (e.g., TREM2 agonists), exosome-based drug delivery systems, and combination therapies. Addressing disease heterogeneity and developing personalized treatments are critical future directions. Ultimately, early interventions aimed at restoring healthy intercellular communication offer new hope for halting AD progression.

## 1 Introduction

The research trajectory of Alzheimer’s disease (AD) has undergone a profound paradigm shift. Early investigations predominantly focused on the amyloid-beta (Aβ) cascade hypothesis, which posits that abnormal Aβ aggregation serves as the central driving force in the pathogenesis of AD (Dong et al., 2012; Ricciarelli and Fedele, 2017). Extensive empirical research has demonstrated that the accumulation of Aβ oligomers and plaques triggers downstream pathological processes, including tau protein hyperphosphorylation, neurofibrillary tangle formation, and synaptic dysfunction (Gulisano et al., 2018; Rajmohan and Reddy, 2017; Wu et al., 2021).However, clinical trials targeting Aβ have repeatedly encountered setbacks, indicating that a singular Aβ-targeted strategy is insufficient to halt or reverse disease progression (Jamal et al., 2025; Jia et al., 2014). These limitations have prompted scientists to re-examine the complex pathological mechanism of AD, recognizing that Aβ deposition represents only a key node in the multi-factor, multi-stage pathological network of AD, rather than the sole causal factor (Jamal et al., 2025).

With the progression of research, the scientific community’s focus has gradually shifted from a singular neuron-centric perspective to the broader domain of neuroimmune interactions. The roles of glial cells, such as microglia and astrocytes, in AD have garnered unprecedented attention (Efthymiou and Goate, 2017; Gao et al., 2022; Gao et al., 2023; Yan et al., 2024; Zhao et al., 2026). Genome-wide association studies (GWAS) have identified numerous risk genes for AD, including TREM2, CD33, and CR1, which are predominantly highly expressed in microglial cells, underscoring the pivotal role of the innate immune system in the pathogenesis of AD (Haure-Mirande et al., 2022; Li et al., 2021; Sudwarts and Thinakaran, 2023). In the brains of AD patients, microglia exhibit dynamic functional state transitions, shifting from a homeostatic phenotype to either disease-associated microglia (DAM) or a neuroinflammatory phenotype (Takatori et al., 2025). This transformation may exert a protective effect by clearing Aβ and cellular debris; however, under conditions of sustained activation, it may also release substantial quantities of pro-inflammatory cytokines, such as IL-1β and TNF-α, thereby exacerbating synaptic damage and neuronal death (Kapoor and Chinnathambi, 2023; Kempuraj et al., 2020; Patel et al., 2025; Zhang et al., 2024a; Zheng et al., 2016). Simultaneously, the dysfunction of metabolic support provided by astrocytes to neurons, coupled with their reactive proliferation, exacerbates neuroinflammation and impairs the functional integrity of neural circuits (Iglesias et al., 2017; Linnerbauer et al., 2020; Patani et al., 2023).

Current research on AD is advancing toward a novel integrative paradigm—the neuroimmune network perspective, which emphasizes the intricate communication mechanisms among diverse cell types. This paradigm conceptualizes neurons, glial cells, the cerebral vascular system, peripheral immune cells, and even the gut microbiota as a highly interconnected functional network (Clemente-Suárez et al., 2023; Toader et al., 2024). Intercellular communication is facilitated through a diverse array of mediators, including cytokines, chemokines, extracellular vesicles (EVs), and the complement system (Berumen Sánchez et al., 2021; Liu and Wang, 2023). For instance, extracellular vesicles (EVs) carrying Aβ and tau proteins facilitate the intercellular transmission of pathological proteins, while the complement pathway (e.g., C1q and C3) is implicated in aberrant synaptic pruning, thereby contributing to early cognitive decline (Cho, 2019; Gomez-Arboledas et al., 2021; Sanfilippo et al., 2025). Furthermore, the infiltration of peripheral immune cells into the brain and the modulation of neuroinflammation by the gut microbiota-brain axis through metabolites such as short-chain fatty acids have significantly expanded the scope of AD pathophysiology (Choi et al., 2022; Junyi et al., 2025).

Consequently, future therapeutic strategies must transcend single-target approaches and shift toward modulating the functional homeostasis of the entire neuroimmune network. By implementing multi-target interventions to restore healthy intercellular communication, these strategies offer novel promise in halting the progression of AD (Sharma et al., 2024; Sharma et al., 2025).

## 2 Cellular basis of neuroinflammation: disruptive factors in brain immune homeostasis

The pathological mechanisms of AD involve extensive dysregulation of the neuroimmune system, wherein the disruption of intercellular communication within the central nervous system(CNS) constitutes one of the pivotal factors driving disease progression, alongside traditional pathways such as Aβ and tau accumulation (Guerriero et al., 2017; Teleanu et al., 2022; Thakur et al., 2023). Neuroinflammation is not solely triggered by the aggregation of Aβ and tau proteins, but also arises from the dysfunction of brain immune cells, including microglia, astrocytes, border-associated immune cells, oligodendrocytes, and endothelial cells of the blood-brain barrier (Liu C. et al., 2025; Wu et al., 2024). These cells accelerate neuronal death through multiple mechanisms: (i) Continuously releasing pro-inflammatory factors such as IL-1β, TNF-α, and reactive oxygen species, which directly damage the integrity of neuronal membranes and mitochondria; (ii) Disrupting synaptic homeostasis by impairing glutamate uptake and promoting complement-mediated excessive synaptic pruning; (iii) Inducing oxidative stress by activating nicotinamide adenine dinucleotide phosphate (NADPH) oxidase and triggering mitochondrial dysfunction. Collectively, these mechanisms create a toxic microenvironment that exacerbates neurodegenerative diseases. (Botella Lucena and Heneka, 2024; Negi and Das, 2020; Wu et al., 2024).

### 2.1 Functional diversity of microglia: from homeostatic state to disease-associated microglia

Microglia, as the resident immune cells of the CNS, play a critical role in maintaining neural homeostasis as part of the brain’s complex regulatory network. Under physiological conditions, they achieve this function through the active clearance of Aβ and apoptotic debris (Kabba et al., 2018; Streit and Xue, 2009; Yin et al., 2017). However, during the early stages of AD, the deposition of Aβ can induce the transformation of microglia into disease-associated microglia (DAM), characterized by diminished phagocytic function and increased secretion of inflammatory factors (Gao et al., 2023; Kim et al., 2022; Ren et al., 2022; Song and Colonna, 2018). DAM cells exhibit elevated expression levels of TREM2 and apolipoprotein E (APOE); however, sustained activation induces lysosomal dysfunction, exacerbating Aβ accumulation (Shi and Holtzman, 2018; Yeh et al., 2016). Genetic research has confirmed that pathogenic variants in the TREM2 gene directly increase the risk of AD by impairing the Aβ clearance capacity of microglia (Colonna and Wang, 2016; Gratuze et al., 2018; Jonsson et al., 2013; Yeh et al., 2016).

Furthermore, the functional heterogeneity of microglia is regulated by the local microenvironment. For instance, microglia surrounding Aβ plaques exhibit activation of the NLRP3 inflammasome, releasing IL-1β and IL-18, thereby promoting the onset of neuroinflammation (Leng and Edison, 2021; Van Zeller et al., 2021). In contrast, microglia distant from plaques may maintain a homeostatic phenotype; however, prolonged exposure to an inflammatory milieu ultimately leads to functional exhaustion (Eggen et al., 2013; Li and Barres, 2018; Woodburn et al., 2021). Single-cell RNA sequencing studies have further elucidated the existence of DAM subpopulations, wherein specific subsets exhibit significant correlations with tau-induced pathogenesis propagation, while other subsets are associated with aberrant synaptic pruning (Hou et al., 2022; Kim et al., 2022; Lee et al., 2022; Preman et al., 2024; Sanfilippo et al., 2025). The interaction between microglia and astrocytes also modulates the transformation of disease-associated microglia (DAM) (Tables 1, 2). In the AD model, tumor necrosis factor-alpha (TNF-α) derived from microglia can induce astrocytes to produce complement protein C1q, thereby enhancing the synaptic phagocytic function of microglia (Huffels et al., 2023; Liang et al., 2023; Schwab and McGeer, 2008). This positive feedback loop accelerates the decline of cognitive functions, while therapies targeting TREM2 or NLRP3 have demonstrated potential in alleviating neuroinflammation in preclinical studies (Ashvin Dhapola et al., 2025; Liu P. et al., 2022; Maurya et al., 2025; Noh et al., 2025). Consequently, modulating the plasticity of microglia constitutes a pivotal therapeutic strategy for AD.

**TABLE 1:** Key neuroimmune-related risk genes in Alzheimer’s disease.

Gene	Risk allele/variant	Proposed pathogenic mechanism in AD
TREM2	R47H, R62H	Loss-of-function mutations impair Aβ clearance and promote a maladaptive DAM phenotype, exacerbating neuroinflammation.
APOE	ε4	The APOE ε4 allele is the prime genetic risk factor for LOAD, driving faster brain atrophy, BBB dysfunction, and cerebral amyloid-β deposition. It also exacerbates tau pathology and impairs Aβ clearance, with its amyloid-β-induced astrocytic expression mediated by the low-density lipoprotein receptor.
**CD33**	rs3865444 (C)	Higher expression increases AD risk by suppressing microglial phagocytosis of Aβ.
CR1	Various SNPs	Alters complement regulation, potentially leading to excessive synaptic pruning and chronic inflammation.
INPP5D (SHIP1)	rs35349669	Dysregulation of this negative regulator may lead to hyperactive microglial responses and increased neuroinflammation.

TREM2, Triggering Receptor Expressed on Myeloid cells 2; APOE, Apolipoprotein E; CD33, Cluster of Differentiation 33; CR1, Complement Receptor 1; INPP5D/SHIP1, Inositol Polyphosphate-5-Phosphatase D/SH2-containing Inositol 5’-Phosphatase 1; AD, Alzheimer’s Disease; SNPs, Single Nucleotide Polymorphisms.

**TABLE 2:** Glial cell states and functions in Alzheimer’s disease.

Cell type	State	Key markers	Core pathogenic role
**Microglia**	Homeostatic	CX3CR1^+^, TREM2low	Physiological surveillance and synaptic pruning.
	**Disease-Associated (DAM)**	TREM2hi, APOEhi, Pro-inflammatory cytokines	**Impaired Aβ clearance, chronic neuroinflammation, synaptic loss.**
**Astrocytes**	Homeostatic	GLT-1 (EAAT2)^+^	Glutamate clearance, neuronal metabolic support.
	**Reactive**	GFAPhi, **C3hi**, S100Bhi	Loss of support function, exacerbation of excitotoxicity and neuroinflammation.

DAM, Disease-Associated Microglia; GFAP, Glial Fibrillary Acidic Protein; GLT-1 (EAAT2), Glutamate Transporter-1 (Excitatory Amino Acid Transporter 2); C3, Complement Component 3; AD, Alzheimer’s Disease; IL-6, Interleukin-6; MCP-1, Monocyte Chemoattractant Protein-1; CX3CR1, CX3C Chemokine Receptor 1; TREM2, Triggering Receptor Expressed on Myeloid cells 2; APOE, Apolipoprotein E.

### 2.2 Reactive astrogliosis: loss of neurosupportive functions and acquisition of toxic effects

Astrocytes within the CNS are responsible for maintaining the integrity of the blood-brain barrier, regulating synaptic transmission, and providing metabolic support (Abbott et al., 2006; Chen Z. et al., 2023; Verkhratsky et al., 2015). In the progression of AD, Aβ protein and inflammatory signaling pathways trigger the reactive activation of astrocytes, resulting in the loss of their supportive functions and the acquisition of neurotoxic properties (Lawrence et al., 2023; Minter et al., 2016). Reactive astrocytes upregulate the expression of glial fibrillary acidic protein (GFAP) and secrete substantial quantities of pro-inflammatory cytokines, including IL-6 and MCP-1, thereby exacerbating the inflammatory cascade (Guo et al., 2014; Johnstone et al., 1999; Jurga et al., 2021; Lu et al., 2019).

This toxic gain manifests as a dysregulation of glutamate metabolism. In AD, astrocytes responsible for clearing synaptic glutamate through glutamate transporters exhibit reduced expression, thereby leading to excitotoxicity and neuronal cell death (Burnyasheva et al., 2023; Massie et al., 2015; Miladinovic et al., 2015; Provenzano et al., 2023). Concurrently, reactive astrocytes produce complement protein C3, which facilitates microglia-mediated excessive synaptic pruning, thereby further impairing memory formation (Chung et al., 2015; Huo et al., 2024; Liu H. et al., 2024; Scott-Hewitt et al., 2023; Soteros and Sia, 2022; Watson and Tang, 2022). Animal model studies have demonstrated that inhibition of the C3 signaling pathway in astrocytes can reverse synaptic loss and cognitive dysfunction (Tan et al., 2023; Zhu et al., 2024).

Metabolic dysregulation also drives the reactivity of astrocytes. Mitochondrial dysfunction in the brains of AD patients induces a shift in astrocytes toward glycolysis, resulting in lactate accumulation and exacerbation of oxidative stress (Newington et al., 2013; Rummel and Butterfield, 2022; Takahashi, 2021). Furthermore, the expression of the APOE ε4 haplotype in astrocytes impairs cholesterol transport, promotes the aggregation of Aβ, and accelerates tau protein phosphorylation (Staurenghi et al., 2022; Sun et al., 2023; Uddin et al., 2019; Zhang et al., 2024b). Therefore, restoring the homeostatic functions of astrocytes may alleviate the pathological progression of AD through multiple therapeutic targets.

### 2.3 Invasion of border immune cells: interaction between peripheral and central immune systems

In the advanced stages of AD, the disruption of the blood-brain barrier facilitates the infiltration of peripheral immune cells, including T cells and monocytes, into the central nervous system. These cells are referred to as border-associated immune cells (Shi M. et al., 2024; Shokr, 2025; Zhang S. et al., 2025). Upon recognition of the Aβ antigen, infiltrating CD4^+^ T cells release IFN-γ, which subsequently activates microglia and amplifies the inflammatory response (Mittal et al., 2019; Monsonego et al., 2006). Concurrently, the impaired functionality of regulatory T cells (Tregs) fails to suppress neuroinflammation, thereby exacerbating the pathological progression (Abdullah et al., 2011; MaruYama et al., 2015; Strutt and Bretscher, 2005). Monocyte-derived macrophages also participate in the clearance of beta-amyloid; however, compared to microglia, their clearance efficiency is relatively lower, and they may secrete more pro-inflammatory factors (Manchikalapudi et al., 2019; Martin et al., 2017; Munawara et al., 2021; Zuroff et al., 2017). In the cerebrospinal fluid of AD patients, elevated levels of neutrophil markers, such as myeloperoxidase (MPO), indicate a widespread activation of the innate immune system (Bawa et al., 2020; Dong et al., 2018). Furthermore, autoantibodies produced by B cells may target neuronal antigens, thereby triggering pathological processes akin to autoimmune responses (López Casado et al., 2018; Rivera-Correa and Rodriguez, 2018; Russo and Lopalco, 2006). These autoantibodies can target a variety of antigens, including Aβ, tau protein, and neuronal surface proteins. Nowadays, there is a growing recognition that they can lead to neurotoxicity and synaptic dysfunction. Similar situations in other neurodegenerative diseases further confirm their pathological role. For instance, in Parkinson’s disease, autoantibodies against α-synuclein have been detected, and these antibodies may affect the aggregation and spread of α-synuclein. Similarly, in multiple sclerosis, B cells become an important factor contributing to pathological changes by producing autoantibodies against myelin components, which indicates the existence of a conserved mechanism of antibody-mediated nerve injury in central nervous system diseases (Beltran-Velasco and Clemente-Suárez, 2025; Giovannini et al., 2021; Kearns, 2024; Russo and Lopalco, 2006).

The gut microbiota modulates the infiltration of marginal immune cells through the gut-brain axis. Studies in AD models demonstrate that gut dysbiosis induces peripheral T-cell activation, thereby increasing the permeability of the blood-brain barrier (Beltran-Velasco and Clemente-Suárez, 2025; Giovannini et al., 2021; Kearns, 2024; Tang et al., 2020; Welcome, 2019). Targeted interventions for the gut microbiota, such as probiotics, have been shown to reduce immune cell infiltration and enhance cognitive function (Białecka-Dêbek et al., 2021; Bonfili et al., 2021). This suggests that modulating the peripheral immune system may emerge as a potential therapeutic strategy for AD.

### 2.4 The role of oligodendrocytes and blood-brain barrier endothelial cells: underestimated key players

Oligodendrocytes are responsible for myelination, ensuring the efficient conduction of neuronal electrical signals. In AD, Aβ and inflammatory factors directly impair the differentiation of oligodendrocyte precursor cells (OPCs), leading to myelin loss and white matter damage (Affrald and Narayan, 2024; Bokulic Panichi et al., 2025; Tylek and Basta-Kaim, 2025; Zou et al., 2023). Postmortem studies have revealed a reduction in the population of OPCs in the brains of AD patients, concomitant with the downregulation of myelin-related gene expression (Lohrberg et al., 2020; Zhou et al., 2022; Zou et al., 2023). This demyelination phenomenon not only decelerates neural conduction velocity but also exacerbates axonal energy stress, thereby accelerating neuronal degeneration (Friese et al., 2014; Stys, 2005).

Endothelial cells of the blood-brain barrier exhibit functional abnormalities during the early stages of AD. Aβ deposition induces the production of reactive oxygen species (ROS) in endothelial cells, disrupts the expression of tight junction proteins (such as claudin-5), and increases barrier permeability (Carrano et al., 2011; Enciu et al., 2013; Erdő et al., 2017; Wan et al., 2014; Yue et al., 2024). This facilitates the infiltration of hematogenous toxins and immune cells into the central nervous system, thereby exacerbating the inflammatory cascade (Cockerill et al., 2018; Kurz et al., 2022; Sweeney et al., 2019). Simultaneously, endothelial cell senescence promotes microglial activation through the secretion of Senescence-Associated Secretory Phenotype (SASP) factors (Carrano et al., 2011; Yue et al., 2024).

The interaction between oligodendrocytes and endothelial cells also exerts a significant influence on the progression of AD. Vascular endothelial growth factor (VEGF) derived from endothelial cells can inhibit the maturation of OPCs, while factors secreted by OPCs regulate vascular stability (Carmeliet and Ruiz de Almodovar, 2013; He et al., 2025; Miyamoto et al., 2014; Ruiz de Almodovar et al., 2009; Sepehrinezhad and Gorji, 2026; Zou et al., 2023). Consequently, targeting these cells may potentially decelerate disease progression by preserving myelin integrity and maintaining the blood-brain barrier. Mounting evidence also suggests that the gut vascular barrier (GVB) may influence the integrity of the blood-brain barrier (BBB). Preliminary data indicate that gut-derived inflammatory factors and microbial metabolites may compromise the gut vascular barrier, thereby increasing the systemic circulation of pro-inflammatory mediators. This may secondarily affect blood-brain barrier function and lead to neuroinflammation. Nevertheless, further mechanistic research on this axis is required in the context of AD (Sweeney et al., 2019; Welcome, 2019).

## 3 Molecular messengers: the core mediators of intercellular communication

**FIGURE 1:**
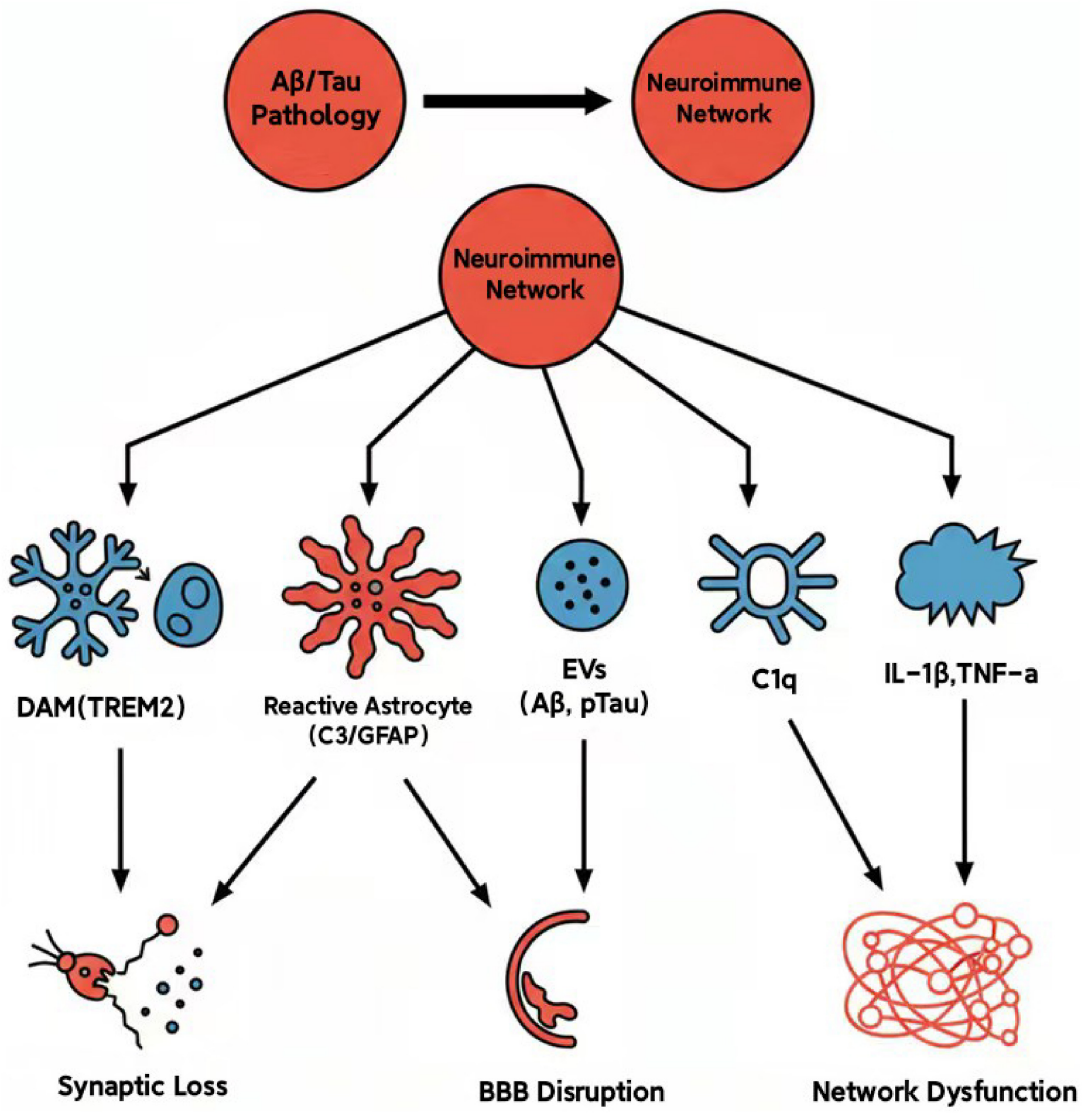
Schematic illustration of the mechanistic role of the neuroimmune network in the core pathogenic processes of Alzheimer’s disease (AD). Amyloid-beta (Aβ) and Tau pathologies, as upstream initiating factors, activate the central neuroimmune network. The core components of this network, including disease-associated microglia (DAM), reactive astrocytes, extracellular vesicles (EVs), complement proteins (e.g., C1q, C3), and inflammatory cytokines, collectively mediate critical pathological events downstream through complex interactions, such as synaptic loss, blood-brain barrier (BBB) disruption, and neural network dysfunction.

The pathological progression of AD is not only characterized by intrinsic metabolic disturbances and protein misfolding within neurons, but also significantly relies on intricate intercellular communication networks. These communication processes are mediated by diverse molecular messengers, including cytokines, chemokines, extracellular vesicles, complement proteins, and damage-associated molecular patterns (DAMPs). Collectively, these molecules establish a disease-specific microenvironment that drives neuroinflammatory responses, pathological protein propagation, and synaptic dysfunction (Figure 1).

### 3.1 Cytokine and chemokine network: construction and maintenance of the inflammatory microenvironment

Cytokines and chemokines play pivotal regulatory roles in neuroimmune interactions by activating microglia and astrocytes, thereby driving chronic neuroinflammation in AD. Pro-inflammatory factors, including interleukin-1β (IL-1β), tumor necrosis factor-α (TNF-α), and interleukin-6 (IL-6), are significantly elevated in the cerebrospinal fluid and brain tissue of AD patients, with their concentrations positively correlating with the degree of cognitive decline (Azizi and Mirshafiey, 2012; Zhang and Jiang, 2015). Cytokines and chemokines play a pivotal regulatory role in neuroimmune interactions by activating microglia and astrocytes, thereby driving chronic neuroinflammation in AD. Pro-inflammatory factors, including interleukin-1β (IL-1β), tumor necrosis factor-α (TNF-α), and interleukin-6 (IL-6), are significantly elevated in the cerebrospinal fluid and brain tissue of AD patients, with their concentrations positively correlating with the degree of cognitive decline (Ali et al., 2024; Al-Kuraishy et al., 2025). Simultaneously, tumor necrosis factor-α (TNF-α) induces neurotoxicity through the TNFR1-mediated signaling pathway, impairing synaptic plasticity; whereas interleukin-1β (IL-1β) suppresses long-term potentiation (LTP), directly affecting memory formation (Bourgognon and Cavanagh, 2020; Levin and Godukhin, 2017; Yirmiya and Goshen, 2011).

Chemokines such as CCL2, CXCL8, and CX3CL1 exacerbate intracerebral inflammatory responses by recruiting peripheral immune cells to cross the blood-brain barrier. The binding of CCL2 to its receptor CCR2 facilitates monocyte infiltration and induces phagocytic dysfunction in microglia (Banisadr et al., 2005; Glabinski et al., 2005; Semple et al., 2010). The CX3CL1-CX3CR1 axis exhibits a dual regulatory function: it maintains microglial quiescence under physiological conditions, while its signaling dysregulation under pathological conditions leads to excessive microglial activation (Mecca et al., 2018; Pawelec et al., 2020). Furthermore, the chemokine network forms a positive feedback loop with Aβ and tau protein pathology: Aβ oligomers stimulate microglia to release CCL3 and CCL5, which in turn exacerbate the hyperphosphorylation of tau protein within neurons (Jorda et al., 2020; Wojcieszak et al., 2022).

Anti-inflammatory cytokines such as IL-4, IL-10, and TGF-β may exert protective effects during the early stages of AD; however, their expression becomes suppressed by the pro-inflammatory microenvironment as the disease progresses. IL-4 facilitates the transformation of microglia into an anti-inflammatory phenotype through the activation of STAT6, thereby enhancing Aβ clearance capacity (Azizi and Mirshafiey, 2012; Rubio-Perez and Morillas-Ruiz, 2012; Zheng et al., 2016). However, the IL-4 signaling pathway in AD is frequently compromised due to receptor downregulation, resulting in the collapse of protective mechanisms (Thakur et al., 2023; Zhao et al., 2015). Therefore, modulating cytokine balance has emerged as a pivotal therapeutic strategy, as evidenced by the remarkable efficacy of anti-TNF-α antibodies or CCR2 antagonists in alleviating cognitive deficits in animal models (Krsek et al., 2024; Moreno et al., 2018).

### 3.2 Extracellular vesicles: transboundary carriers of pathological proteins and genetic information

Extracellular vesicles (EVs), including exosomes and microvesicles, are key mediators of intercellular communication, facilitating the transport of bioactive molecules such as proteins, lipids, and nucleic acids. In AD, EVs secreted by neurons, microglia, and astrocytes are closely related to the propagation of Aβ and tau proteins. Aβ oligomers can be encapsulated into vesicles by binding to phosphatidylserine on the EV surface and then be transferred between cells via EVs (Meldolesi, 2021; Paolicelli et al., 2019; Pegtel et al., 2014). Similarly, hyperphosphorylated tau proteins can be transported via neuronal extracellular vesicles (EVs) and internalized into adjacent cells through endocytosis, thereby inducing template-directed protein aggregation (Cheng H. B. et al., 2023).

Extracellular vesicles (EVs) exhibit cell type-specific cargo compositions, rendering them potential biomarkers for disease diagnosis. Notably, neuron-derived extracellular vesicles are enriched with neurofilament light chain (NfL) and tau proteins, whose concentrations demonstrate significant correlations with the degree of brain atrophy (Si et al., 2023). Microglial extracellular vesicles (EVs) transport inflammatory mediators, including IL-1β and complement components, which serve as biomarkers for the level of neuroinflammation (Raffaele et al., 2020; Yang et al., 2018). Recent studies have demonstrated a significant increase in the quantity of GFAP-positive astrocyte-derived extracellular vesicles (EVs) in the plasma of patients with AD, which exhibits a positive correlation with Aβ deposition (Forró et al., 2024; Koivumäki et al., 2024).

Extracellular vesicles (EVs) regulate recipient cell functions through the delivery of non-coding RNAs. Microglia-derived extracellular vesicles contain miR-155 and miR-146a, which can be internalized by neurons, thereby suppressing the expression of synapse-associated genes (Huang et al., 2025a). Conversely, neuron-derived miR-124 can suppress microglial activation through extracellular vesicles (EVs); however, this regulatory mechanism is impaired in AD (Gong et al., 2025; Xu et al., 2022). Furthermore, extracellular vesicles (EVs) possess the capability to traverse the blood-brain barrier, thereby facilitating the transmission of central nervous system signals to the peripheral immune system. This phenomenon may elucidate the pathogenesis of systemic inflammatory responses in AD (Cabrera-Pastor, 2024; Matsumoto et al., 2017).

In the therapeutic domain, engineered extracellular vesicles (EVs) have emerged as a novel drug delivery platform. Mesenchymal stem cell-derived EVs loaded with BACE1 siRNA have demonstrated the capability to reduce Aβ production and ameliorate memory deficits in AD mouse models (Yin et al., 2023; Zhou et al., 2024). Similarly, extracellular vesicles (EVs) carrying anti-inflammatory microRNAs (miRNAs) can inhibit the activation of microglial cells, thereby alleviating neuroinflammatory responses (Ghosh and Pearse, 2024; Prada et al., 2018). However, the heterogeneity of extracellular vesicles and their uptake efficiency remain significant challenges in the process of clinical translation (Ghodasara et al., 2023; Song et al., 2022).

### 3.3 The complement system: transition from physiological synaptic pruning to pathological phagocytosis

The complement system serves as a pivotal component of innate immunity, mediating physiological synaptic pruning during development, while its dysfunction contributes to pathological synaptic loss in AD. As key initiators of the complement cascade, C1q and C3 exhibit selective perisynaptic deposition in the brains of AD patients (Gomez-Arboledas et al., 2021; Zhong et al., 2023). Aβ oligomers can activate C1q, thereby initiating the classical pathway, leading to the deposition of C3b on synaptic surfaces, which subsequently marks these synapses for microglial phagocytosis (Guan et al., 2023; Webster et al., 2000). Furthermore, tau protein can directly activate the complement system by binding to C1q, a process that operates independently of the Aβ pathway (Guan et al., 2023; Morgan, 2018).

Microglia express complement receptors CR3 and CR4, which initiate the phagocytic process upon recognition of bound C3b. In AD models, inhibition of CR3 has been demonstrated to reduce synaptic loss and improve cognitive function (Crehan et al., 2012; Han Q. Q. et al., 2024). However, complement activation also exhibits protective effects: C5a enhances microglial phagocytosis of Aβ through its receptor C5aR1, whereas excessive activation can lead to inflammatory damage (Carvalho et al., 2022; Hernandez et al., 2017; Li et al., 2023). This duality renders complement regulation a highly precise therapeutic target.

Complement regulatory proteins such as CD59 and CFH exhibit aberrant expression in AD, thereby accelerating complement activation. CD59 functions to inhibit the formation of the membrane attack complex (MAC), safeguarding neurons from lytic damage; however, in the brains of AD patients, CD59 has been found to be downregulated by Aβ (Akhtar-Schäfer et al., 2018). CFH exerts its regulatory function by inhibiting the alternative pathway of complement activation, and its gene polymorphism is associated with the risk of AD (Lukiw and Alexandrov, 2012; Zetterberg et al., 2008). Recent research has further elucidated the direct correlation between the complement system and synaptic plasticity: C3-deficient mice exhibit enhanced LTP and improved memory capabilities (Shi et al., 2015).

The therapeutic strategies targeting the complement system encompass C1q antibodies, C3 inhibitors, and C5aR antagonists. In APP/PS1 mouse models, anti-C1q antibody treatment has been shown to mitigate synaptic loss and restore network activity. Therapeutic strategies targeting the complement system encompass C1q antibodies, C3 inhibitors, and C5aR antagonists. In the APP/PS1 mouse model, anti-C1q antibody treatment has been demonstrated to mitigate synaptic loss and restore network activity (Daskoulidou et al., 2025; Schartz and Tenner, 2020). However, prolonged suppression of the complement system may elevate the risk of infections, suggesting that localized or intermittent intervention could represent a safer alternative approach However, prolonged suppression of the complement system may elevate the risk of infections, suggesting that localized or intermittent intervention could represent a safer alternative approach (Mastellos et al., 2019; Ram et al., 2010).

### 3.4 Damage-associated molecular patterns: endogenous danger signals activating innate immunity

Damage-associated molecular patterns (DAMPs) are endogenous molecules released following cellular stress or death, which activate innate immune responses through pattern recognition receptors (PRRs). In AD, Aβ and tau proteins function as DAMPs, binding to TLR2, TLR4, and RAGE receptors, thereby inducing microglial activation (Roh and Sohn, 2018; Venegas and Heneka, 2017). Furthermore, classical damage-associated molecular patterns (DAMPs), including mitochondrial DNA, adenosine triphosphate (ATP), and high-mobility group box 1 (HMGB1), exhibit significantly elevated levels in the brains of patients with AD.

HMGB1 is released by necrotic neurons, binds to TLR4 and RAGE, and facilitates the assembly of the NLRP3 inflammasome as well as the maturation of IL-1β (Behl et al., 2021; Nogueira-Machado et al., 2011). Inhibition of high mobility group box 1 protein alleviates neuroinflammation and ameliorates cognitive dysfunction (Kong et al., 2017; Tan et al., 2021). Mitochondrial DNA activates microglia through TLR9, and its release is closely associated with mitochondrial dysfunction (Moya et al., 2021; Pinti et al., 2021). ATP facilitates the activation of NLRP3 through the P2X7 receptor, thereby completing the assembly of the inflammasome (Franceschini et al., 2015; Wang et al., 2020).

Damage-associated molecular patterns (DAMPs) are directly involved in the pathological protein aggregation process. Research indicates that high mobility group box 1 protein (HMGB1) can bind to Aβ, thereby enhancing its oligomerization and neurotoxicity (Kaur et al., 2023; Venegas and Heneka, 2017). S100B, as an astrocyte-derived damage-associated molecular pattern (DAMP), not only promotes inflammatory responses but also directly upregulates the expression of BACE1, thereby accelerating the production of Aβ (Sarkar et al., 2025; Yue and Hoi, 2023). These interactions establish a positive feedback loop between damage-associated molecular patterns (DAMPs) and pathological proteins.

Targeting the damage-associated molecular patterns (DAMPs) signaling pathway has emerged as a potential therapeutic strategy. Anti-high mobility group box 1 (HMGB1) antibodies, Toll-like receptor 4 (TLR4) antagonists, and P2X7 inhibitors have demonstrated significant efficacy in preclinical models (Andersson et al., 2021; Ren et al., 2023; Xue et al., 2021). However, damage-associated molecular patterns (DAMPs) also play a pivotal role in physiological processes. For instance, high-mobility group box 1 (HMGB1) is involved in DNA repair, and complete inhibition of its function may lead to adverse consequences (Lin et al., 2025; Pandolfi et al., 2016). Therefore, modulation may constitute a more rational strategic approach compared to complete inhibition.

## 4 Mechanisms of interaction in core pathophysiological processes: from aβ generation to tau protein propagation

### 4.1 Neuroimmune interactions modulate aβ pathology: dysregulation of microglial clearance function and neuronal stress responses

Microglia, as the main immune cells in the central nervous system, play a crucial role in the clearance of Aβ. Their dysfunction directly leads to Aβ deposition, a process regulated by multiple signaling pathways. The phagocytic function mediated by TREM2 is essential for Aβ clearance, and its pathogenic variant significantly increases the risk of AD (Lee et al., 2025; Xiang et al., 2016). The APOE isoforms influence the clearance efficiency of Aβ through the modulation of microglial metabolic reprogramming, with APOE ε4 carriers exhibiting the most adverse outcomes (Raulin et al., 2022; Yamazaki et al., 2019).

Mitochondrial dysfunction exacerbates the decline in microglial phagocytic capacity. Microglia in AD patients exhibit increased mitochondrial fragmentation and insufficient energy production (Ashleigh et al., 2023; Li Y. et al., 2022). This metabolic deficiency is further exacerbated through the mTOR signaling pathway, resulting in the disruption of autophagic flux and the abnormal accumulation of Aβ protein (Cheng L. et al., 2023; de la Monte, 2023).

Neuronal stress responses and microglial activation form a vicious cycle. Aβ oligomers induce endoplasmic reticulum stress in neurons, leading to the release of damage-associated molecular patterns (DAMPs) (Lin et al., 2022; Salminen et al., 2009). These signals activate microglia through the TLR4/MyD88/NF-κB signaling pathway, thereby triggering a burst release of inflammatory cytokines (Li Z. et al., 2022; Zhu et al., 2014). Activated microglia further release reactive oxygen species, leading to neuronal oxidative damage and increased production of Aβ protein (Qin et al., 2002; Schilling and Eder, 2011).

The gut microbiota-gut-brain axis influences Aβ pathology through immune modulation. Metabolites of the gut microbiota, particularly short-chain fatty acids, regulate the maturation and function of microglia (Cao et al., 2025; Qian et al., 2022). Microbial dysbiosis facilitates the entry of inflammatory cytokines into systemic circulation, compromises the integrity of the blood-brain barrier, and exacerbates neuroinflammatory responses (Beltran-Velasco and Clemente-Suárez, 2025; Welcome, 2019).

### 4.2 Neuroinflammation drives tau hyperphosphorylation and propagation: the pivotal role of glial cells

Neuroinflammation establishes a conducive microenvironment for the hyperphosphorylation of tau protein. Microglia-derived IL-1β and TNF-α activate the intraneuronal kinase system, including GSK-3β and CDK5 (Botella Lucena and Heneka, 2024; Kakkar et al., 2025). These kinases phosphorylate specific sites on the tau protein, thereby reducing its binding affinity to microtubules and increasing its propensity for aggregation (Haj-Yahya et al., 2020; Liu et al., 2007).

Astrocytes play a pivotal role in the propagation of tau protein pathology. Reactive astrocytes release vesicles containing tau seeds, thereby facilitating their dissemination through the extracellular space (Polanco and Götz, 2022; Ruan, 2022). These tau seeds are internalized into healthy neurons via clathrin-mediated endocytosis, subsequently inducing the aggregation of endogenous tau proteins (Hivare et al., 2023; Zhang et al., 2024c).

The complement system-mediated synaptic pruning accelerates the progression of tau-induced pathogenesis. C1q and C3 deposits on the surface of hyperphosphorylated tau neurons, marking these cells for microglial phagocytosis (Heppner et al., 2015; Jiang and Bhaskar, 2017). This aberrant synaptic pruning leads to synaptic loss and facilitates the release of tau proteins into the extracellular space (Vogels et al., 2019; Wu et al., 2021).

Alterations in the extracellular matrix facilitate the propagation of tau proteins. The enhanced activity of matrix metalloproteinases (MMPs) compromises the structural integrity of the extracellular matrix, thereby creating pathways for the dissemination of tau proteins (Pintér and Alpár, 2022; Radosinska and Radosinska, 2025). In particular, MMP-9 enhances the permeability of the blood-brain barrier by degrading tight junction proteins, thereby facilitating the extravasation of tau protein into the peripheral circulation (Spampinato et al., 2017; Weekman and Wilcock, 2016).

### 4.3 Immunological mechanisms of synaptic pruning: the role of complement C1q in synaptic plasticity in the brain

Complement-mediated synaptic pruning constitutes a pivotal early event in the pathogenesis of AD. The deposition of C1q on the presynaptic membrane triggers the classical complement cascade, ultimately leading to the deposition of C3 opsonin (Gomez-Arboledas et al., 2021; Perry and O’Connor, 2008). Microglia recognize C3 fragments via the CR3 receptor and phagocytose the labeled synaptic structures (Fu et al., 2012; Han Q. Q. et al., 2024).

The relationship between gender and complement-mediated synaptic loss in AD is complex and appears to involve counterbalancing mechanisms. Although AD is more prevalent in women, potentially due to factors such as longer lifespan and the loss of neuroprotective effects of estrogen after menopause, specific biological mechanisms may confer relative advantages under certain conditions. For instance, estrogen can regulate the expression levels of microglial CR3, a mechanism that may contribute to modulating synaptic pruning prior to or during the early stages of menopause (Crespo-Castrillo and Arevalo, 2020; Price et al., 2025). Additionally, the higher expression levels of X chromosome-encoded complement regulatory proteins in females might offer a protective advantage by fine-tuning complement activity (Bianchi et al., 2012; Libert et al., 2010). However, these potential protective mechanisms are likely insufficient to fully counteract the overall increased risk and pathological drivers in females, particularly in the context of APOE ε4 carriage and postmenopausal endocrine changes. This underscores the multifactorial nature of AD risk, where protective factors at one level may be overwhelmed by risk factors at another.

The APOE haplotype exerts a regulatory influence on the intensity of synaptic pruning. In individuals carrying the APOE ε4 allele, microglia exhibit excessive phagocytic activity, resulting in premature synaptic loss (Chung et al., 2016; Wang et al., 2021). APOE2 safeguards synaptic integrity by upregulating the expression of C1q inhibitory factors (Guan et al., 2023; Yin et al., 2019).

Therapeutic strategies targeting the complement pathway demonstrate significant potential. Anti-C1q antibodies effectively reduce synaptic loss and enhance cognitive function (Dalakas et al., 2020; Tenner and Petrisko, 2025). C3a receptor antagonists effectively preserve synaptic plasticity by inhibiting inflammatory signaling pathways (Pekna et al., 2021; Stokowska et al., 2017).

### 4.4 Dysfunction of vascular units: the vicious cycle of neurovascular coupling and immune cell infiltration

The disruption of the blood-brain barrier constitutes a pivotal hallmark in the vascular pathology of AD. The degeneration of pericytes leads to the downregulation of tight junction proteins, particularly claudin-5 and occludin (Kook et al., 2013; Scalise et al., 2021). The thinning of the basement membrane, concomitant with the degradation of type IV collagen, results in increased vascular permeability (Sage, 1982; Thomsen et al., 2017).

Neurovascular uncoupling impairs energy metabolism. The attenuation of vasodilatory responses during heightened neuronal activity results in insufficient glucose supply (Drew, 2022; Phillips et al., 2016). Dysfunction of the nitric oxide pathway serves as the primary etiology, predominantly manifested by a significant reduction in endothelial nitric oxide synthase activity (Atochin and Huang, 2010).

Peripheral immune cell infiltration exacerbates neuroinflammatory responses. Neutrophils degrade the basement membrane through matrix metalloproteinase-9 (MMP-9), thereby compromising the integrity of the blood-brain barrier (Huang et al., 2021; Rosell et al., 2008; Walz and Cayabyab, 2017). Monocyte-derived macrophages differentiate into inflammatory phenotypes within the brain, releasing interleukin-1β and tumor necrosis factor-α (Tedesco et al., 2015; Zhao et al., 2020).

The impaired perivascular space drainage function facilitates the pathological accumulation of proteins. Lymphatic system dysfunction significantly reduces the efficiency of Aβ clearance (Chachaj et al., 2023; Kylkilahti et al., 2021). The attenuation of arterial pulsation and the impairment of aquaporin-4 polarization in astrocytes constitute the primary etiological factors (Iacovetta et al., 2012; Wolburg et al., 2009).

The VEGF signaling pathway exerts a dual regulatory effect on vascular integrity. Physiological concentrations of VEGF promote endothelial cell survival, whereas elevated concentrations enhance vascular permeability (Ahmad and Nawaz, 2022; Byrne et al., 2005; Zachary, 2001). VEGFR2 inhibitors have been demonstrated to enhance the functionality of the blood-brain barrier, notwithstanding their potential implications on angiogenesis (Liu J. et al., 2020; Zhang et al., 2023).

## 5 Frontier perspectives and emerging technologies: decoding complex interaction networks

The pathological mechanisms of AD are increasingly recognized as a dynamic network involving multiple cell types, signaling pathways, and molecular events. Traditional research has primarily focused on the accumulation of amyloid-β and tau proteins; however, recent advancements have highlighted the critical roles of neuroimmune interactions, intercellular communication, and systemic regulation in the onset and progression of the disease. The integration of spatial omics and single-cell technologies, advanced model systems, emerging signaling pathways, and systems biology provides a comprehensive framework for elucidating the core mechanisms of this complex interaction network.

### 5.1 Spatial omics and single-cell technologies: unveiling the spatiotemporal dynamics of cellular interactions

The advancements in single-cell RNA sequencing (scRNA-seq) and spatial transcriptomics technologies have enabled researchers to analyze cellular heterogeneity and spatial interactions in AD brain tissues at single-cell resolution (Piwecka et al., 2023; Zhang et al., 2024d). These advanced technologies not only elucidate the dynamic changes of neurons, glial cells, and immune cells during disease progression but also identify novel cellular subtypes and their functional state transitions (Piwecka et al., 2023; Zhang et al., 2024d). Recent studies have identified multiple functional subpopulations of microglia in AD, wherein certain subtypes exhibit elevated expression of inflammation-related genes (e.g., TREM2, C1q) and demonstrate close co-localization with Aβ plaques, suggesting their involvement in the clearance of pathological proteins and the modulation of neuroinflammation (Liang X. et al., 2021; Liu et al., 2023). By integrating scRNA-seq and spatial transcriptomic data, researchers have successfully constructed spatiotemporal dynamic maps of cellular interactions within brain regions, thereby elucidating the communication patterns among various cell types across different stages of disease progression (Longo et al., 2021; Piwecka et al., 2023; Xia et al., 2025).

Furthermore, single-cell multi-omics technologies, such as concurrent transcriptomic and epigenomic analyses, have significantly advanced our understanding of cell-specific mechanisms in AD (Baysoy et al., 2023; Chen C. et al., 2023). For instance, a study conducted on human brain tissue samples revealed that DNA methylation patterns in oligodendrocyte precursor cells (OPCs) undergo alterations during the early stages of AD. These epigenetic modifications may potentially impact their differentiation and myelin maintenance functions, thereby exacerbating neuronal damage (Egawa et al., 2019; Tiane et al., 2019). Spatial omics technologies have further elucidated the region-specific distribution patterns of Aβ and tau pathologies, as well as the intricate associations between these pathologies and local immune responses (Maïer et al., 2023; Walker et al., 2024; Xia et al., 2025). For instance, the activation states of microglia in the hippocampal and cortical regions exhibit significant disparities, which may elucidate the heightened susceptibility of these areas during the initial phases of disease progression (Correale, 2014; Leng and Edison, 2021). These research findings not only provide novel insights into the spatial heterogeneity of AD pathology but also establish a theoretical foundation for the development of region-specific therapeutic strategies (Mohanty et al., 2023; Wang M. et al., 2016; Zhang D. et al., 2025).

Despite the unprecedented resolution offered by these technologies, their application continues to encounter significant challenges, including sample preservation, data integration, and the complexity of computational analysis (Dezem et al., 2024). In the future, the integration of high-resolution imaging technologies with artificial intelligence-assisted data analysis methodologies will significantly enhance our understanding of the cellular interaction networks in AD (Liu C. et al., 2025).

### 5.2 Advanced model systems: from human-induced pluripotent stem cell-derived 3D organoids to *in vivo* imaging technologies

Traditional animal models, such as transgenic mice, exhibit limitations in replicating the complex pathology of AD, particularly in fully recapitulating the unique cell types and pathological features characteristic of the human brain (Myers and McGonigle, 2019; Polis and Samson, 2024). Human induced pluripotent stem cell (iPSC)-derived three-dimensional brain organoid models have partially addressed this issue, demonstrating their capability to recapitulate human brain cellular diversity, structural organization, and pathological progression (Lee et al., 2017; Luo et al., 2021; Miyake and Shimada, 2022). For instance, research utilizing iPSC-derived organoid models from AD patients has successfully recapitulated key pathological features, including Aβ deposition, tau protein phosphorylation, and neuroinflammation, as evidenced by the upregulation of pro-inflammatory cytokines (e.g., IL-1β, TNF-α), activation of microglial and astrocytic markers (e.g., IBA1, GFAP), and increased expression of inflammasome components such as NLRP3 (Qian and Tcw, 2021; Yanakiev et al., 2022). This provides a crucial platform for screening drugs targeting neuroimmune interactions.

### 5.3 *In vivo* imaging technologies for dynamic pathological observation

Advancements in *in vivo* imaging technologies, such as two-photon microscopy and positron emission tomography (PET) imaging, have enabled researchers to observe the pathological dynamics in AD models in real time (Pallen et al., 2021). For instance, by utilizing transgenic animals expressing fluorescent reporter genes, researchers have visualized the interaction process between microglia and Aβ plaques, revealing that microglia participate in the dynamic clearance of plaques through a “phagocytosis-exocytosis” cycle (Wendt et al., 2022; Zhao et al., 2017). Furthermore, novel positron emission tomography (PET) probes, such as TSPO-targeting tracers, have enabled non-invasive clinical monitoring of neuroinflammation, thereby providing invaluable tools for disease staging and therapeutic evaluation (Liu Y. et al., 2025; Van Camp et al., 2021).

The integration of organoid models with *in vivo* imaging technologies is propelling AD research toward a more human-relevant and dynamic paradigm (Han X. et al., 2024; Shi H. et al., 2024). For instance, the transplantation of organoids into murine brains, coupled with longitudinal imaging techniques to observe their interactions with host cells, has established an innovative platform for investigating human cellular behavior in *in vivo* environments (Chiaradia and Lancaster, 2020; Kelley et al., 2024; Mansour et al., 2018). However, organoid models still face challenges such as insufficient vascularization and limited maturity. In the future, the development of more complex multicellular organoids (e.g., those incorporating microglia and vascular structures) and microfluidic organ-on-a-chip systems holds promise for better simulating the physiological and pathological environments of the human brain (Fan et al., 2025).

### 5.4 Emerging signaling pathways: the roles of cGAS-STING, ZBP1, and inflammasomes in AD

Recent research has elucidated the pivotal roles of multiple innate immune signaling pathways in AD, including the cGAS-STING pathway, ZBP1, and the inflammasome pathway (Quan et al., 2025; Zhan et al., 2024). The cGAS-STING signaling pathway is typically activated in response to cytoplasmic DNA, such as mitochondrial DNA or viral DNA, thereby driving type I interferon responses and neuroinflammatory reactions (Huang et al., 2023; Paul et al., 2021; Quan et al., 2025). In AD, the accumulation of Aβ and neuronal damage may lead to the leakage of mitochondrial DNA into the cytoplasm, thereby activating the cGAS-STING pathway, which subsequently promotes inflammatory responses in microglia and astrocytes (Liu J. et al., 2024; Quan et al., 2025). Inhibition of this pathway has been demonstrated to alleviate neuroinflammation and improve cognitive function in AD models, indicating its potential therapeutic value as a target (Dhapola et al., 2021; Liu P. et al., 2022).

ZBP1 (Z-DNA Binding Protein 1) represents another molecular entity involved in the regulation of cellular death and inflammatory processes (Huang et al., 2025b; Szczesny et al., 2018). The research findings indicate that ZBP1 may participate in the process of pyroptosis in AD by sensing changes in nucleic acid structures, thereby exacerbating neuronal damage (Guo et al., 2023; Zheng and Kanneganti, 2020). The activation of inflammasomes, such as NLRP3, constitutes a pivotal mechanism underlying neuroinflammation in AD (Shen et al., 2020; Zhang et al., 2020). Aβ fibers and tau oligomers can activate the NLRP3 inflammasome, leading to the maturation and release of IL-1β and IL-18, thereby amplifying the inflammatory response and compromising the blood-brain barrier (Beder et al., 2024; Heneka et al., 2018; Van Zeller et al., 2021). Inhibitors targeting NLRP3 have demonstrated protective effects in preclinical studies; however, challenges pertaining to target specificity and safety profiles remain to be addressed (Coll et al., 2015; Schwaid and Spencer, 2021).

These pathways do not operate in isolation; rather, they constitute an intricate interactive network (Decout et al., 2021; Liu J. et al., 2024; Zhan et al., 2024). For instance, activation of the cGAS-STING pathway may potentiate the assembly of the NLRP3 inflammasome, while ZBP1 could synergistically promote cell death in conjunction with mitochondrial dysfunction (Lei et al., 2023; Murthy et al., 2020; Zhan et al., 2024). Comprehending the intricate interplay among these pathways is paramount for devising synergistic intervention strategies (Liu J. et al., 2024; Murthy et al., 2020).

### 5.5 Systems biology integration: from “key driver genes” to network pharmacology

Systems biology approaches, encompassing network analysis and multi-omics integration, are revolutionizing our comprehension of AD mechanisms and the development of therapeutic strategies (Clark et al., 2021; Rahimzadeh et al., 2024). By integrating genomic, transcriptomic, proteomic, and metabolomic data, researchers are able to identify the “key driver genes” and core regulatory networks in AD (Bertrand et al., 2015; Cheng J. et al., 2023). For instance, network analysis based on large-scale human brain datasets reveals that genes such as APOE, TREM2, and INPP5D occupy central positions within the immunometabolic network, with their variations significantly impacting microglial function and disease risk (Liu T. et al., 2020).

Network pharmacology further leverages these findings to devise multi-target therapeutic strategies. For instance, in addressing neuroimmune interactions and metabolic dysregulation in AD, researchers have proposed a combinatorial approach utilizing pathway inhibitors, which concurrently modulates microglial activation, mitochondrial function, and insulin signaling (Erichsen and Craft, 2023; Li Y. et al., 2022; Suresh et al., 2021). Artificial intelligence-assisted drug repositioning analysis has identified multiple approved pharmaceuticals, including antidiabetic and anti-inflammatory agents, which may exhibit neuroprotective effects through mechanisms involving multi-target regulation (Roix et al., 2014; Schein, 2021).

However, systems biology approaches are confronted with significant challenges, encompassing data heterogeneity, model complexity, and difficulties in clinical translation (Angione, 2019; Wolkenhauer et al., 2013). In the future, the establishment of larger-scale multi-omics databases, the development of more precise computational models, and the advancement of experimental validation technologies will significantly accelerate the translation of these research findings into clinical applications (Doran et al., 2021).

## 6 Therapeutic prospects and future development directions

### 6.1 Immunomodulatory therapy: from broad-spectrum anti-inflammatory to precision targeting

Traditional anti-inflammatory agents, such as nonsteroidal anti-inflammatory drugs (NSAIDs), have demonstrated limited efficacy in clinical trials for AD, which can be partially attributed to their broad-spectrum activity and nonspecific immunosuppressive effects (Jennings et al., 2021; Saliev and Singh, 2025). In recent years, research focus has shifted toward precisely targeting key regulatory factors of innate and adaptive immunity (Hillion et al., 2020; Wang R. et al., 2024). For instance, TREM2 agonist antibodies can enhance the phagocytic function of microglia and promote their transformation into a neuroprotective phenotype, thereby reducing Aβ plaque accumulation in animal models and improving cognitive function (Fassler et al., 2021; Schlepckow et al., 2023; Zhao et al., 2022). The complement system is aberrantly activated in AD, leading to excessive synaptic pruning and the onset of neuroinflammation; preclinical studies have demonstrated that anti-C1q or C3a receptor antagonists exert protective effects on blood-brain barrier integrity and reduce neuronal loss (Schartz and Tenner, 2020; Tenner and Petrisko, 2025). Furthermore, monoclonal antibodies targeting pro-inflammatory cytokines, such as IL-1β and IL-6, are transitioning from trials in rheumatic diseases to AD studies. The objective is to specifically inhibit neuroinflammation without inducing systemic immunosuppression (Garmendia et al., 2024; Markovics et al., 2021). These strategies signify an evolution from broad-spectrum anti-inflammatory approaches to precise immune modulation (Table 3).

**TABLE 3:** Emerging neuroimmune-targeted therapies for Alzheimer’s Disease.

Therapeutic target	Representative agent(s)	Mechanism and key challenge
TREM2 Agonism	AL002a	Mechanism: Activates microglia to enhance Aβ clearance. Challenge: Defining therapeutic time window.
Complement (C1q)	ANX005	Mechanism: Blocks pathological synaptic pruning. Challenge: Risk of immunosuppression.
IL-1β Pathway	Canakinumab	Mechanism: Neutralizes key inflammatory cytokine. Challenge: Systemic immunosuppression.
cGAS-STING	H-151	Mechanism: Inhibits neuroinflammatory signaling driven by mtDNA. Challenge: Intracellular target accessibility.

TREM2, Triggering Receptor Expressed on Myeloid cells 2; C1q, Complement Component 1q; IL-1β, Interleukin-1 beta; cGAS-STING, Cyclic GMP-AMP Synthase—Stimulator of Interferon Genes; AD, Alzheimer’s Disease; Aβ, Amyloid-beta.

### 6.2 Revolutionizing intercellular communication: exosome-based strategies for drug delivery and gene therapy

Exosomes, as natural nanocarriers, have emerged as a promising strategy to specifically modulate neuroinflammation in AD. Their innate ability to cross the blood-brain barrier and target specific cell types, particularly microglia and astrocytes, makes them ideal for delivering anti-inflammatory therapeutics directly to the core of the pathological immune response (Heidarzadeh et al., 2021; Iqbal et al., 2024; Sun et al., 2025). Engineered exosomes can be loaded with anti-inflammatory cargo such as small interfering RNA (siRNA) to silence key pro-inflammatory genes (e.g., NLRP3, IL-1β), microRNAs (e.g., miR-124, miR-146a) to repolarize microglia toward a protective phenotype, or anti-inflammatory cytokines (e.g., IL-10) to counteract the chronic inflammatory milieu in the AD brain (Liang Y. et al., 2021; Ortega et al., 2020).

For instance, exosomes derived from mesenchymal stem cells (MSCs) overexpressing IL-10 have been shown to significantly reduce levels of pro-inflammatory factors like TNF-α and IL-6, alleviate microglial activation, and improve cognitive function in AD models (Lin et al., 2024; Liu S. et al., 2022). Similarly, exosomes loaded with anti-Aβ siRNA not only reduce the amyloidogenic process but also concurrently attenuate the associated neuroinflammatory response, demonstrating a dual benefit (Liu S. et al., 2022; Wang C. et al., 2024; Zhu et al., 2023). The specificity of exosomes can be further enhanced by surface modification with targeting ligands (e.g., TREM2-specific peptides) to achieve precise delivery to disease-associated microglia (DAM), thereby maximizing therapeutic efficacy while minimizing off-target effects (Zhu et al., 2023). Despite their potential, challenges such as exosome heterogeneity, scalable production, and standardized drug loading efficiency remain significant hurdles for clinical translation (Ghodasara et al., 2023; Song et al., 2022). Future efforts should focus on optimizing exosome engineering to develop robust, inflammation-targeted nanotherapeutics for AD.

Gene therapy strategies also employ viral vectors such as adeno-associated virus (AAV) to deliver protective genes; the AAV-encoded TREM2 variant enhances microglial clearance capacity and mitigates tau-induced pathogenesis in the APP/PS1 mouse model (Chira et al., 2015; Fol et al., 2016). These methodologies offer precise interventions targeting the underlying causes of diseases by reshaping cellular communication networks.

### 6.3 Emerging paradigm in combination therapy: dual targeting of pathological proteins and neuroimmune pathways

Monotherapy often proves inadequate in addressing the multifactorial pathological mechanisms of AD, thereby establishing combination therapy as an emerging trend in treatment strategies (Gong et al., 2018; Shirbhate et al., 2022). For instance, the combined application of Aβ monoclonal antibodies (e.g., aducanumab) and TREM2 agonists has demonstrated synergistic effects in animal models: the former facilitates the clearance of existing plaques, while the latter enhances the sustained surveillance capacity of microglia (Anitha et al., 2025; Decourt et al., 2022; Topalis et al., 2025). Similarly, the combined administration of tau protein aggregation inhibitors and IL-1β antagonists concurrently mitigates neurofibrillary tangles and neuroinflammation, thereby enhancing cognitive function (Chen and Yu, 2023; Gaikwad et al., 2024). Furthermore, the combined application of metabolic modulators (such as metformin) and immunotherapy has effectively addressed the concurrent issues of energy metabolism defects and immune dysregulation in AD (Gaikwad et al., 2024; Hua et al., 2023). The combined strategy necessitates optimization of dosage and temporal windows to maximize therapeutic efficacy while minimizing adverse effects.

### 6.4 Challenges and future directions: personalized therapy, biomarkers, and clinical trial design

The implementation of these strategies faces multifaceted challenges. Primarily, the heterogeneity of AD necessitates the adoption of personalized treatment approaches: APOE ε4 carriers may derive greater benefits from immunomodulatory therapies, whereas the tau protein-dominant subtype requires a primary emphasis on anti-tau therapeutics (Butt et al., 2022; Dincer et al., 2022). The development of biomarkers holds paramount significance (Table 4), with neuroinflammation PET imaging (TSPO ligands) and blood levels of GFAP and sTREM2 being particularly prominent. These biomarkers are critical for patient stratification in clinical trials and for monitoring responses to investigational therapies targeting neuroimmune pathways (e.g., TREM2 agonists or anti-inflammatory agents), even in the absence of currently approved disease-modifying therapies (Lista et al., 2024; Werry et al., 2019; Yasuno et al., 2022). However, interpreting these neuroimmune-related biomarkers requires a cautious and multifaceted approach, as recently underscored by Bettcher et al. (2025). First, a single inflammatory marker is unlikely to capture the complexity of the entire neuroimmune cascade; thus, future studies should prioritize measuring a panel of markers with distinct or complementary functions (e.g., combining glial activation markers like GFAP with microglial response markers like sTREM2 and complement proteins). Second, while human association studies have identified correlations, they are insufficient to infer causality; mechanistic validation in experimental models remains crucial. Third, neuroinflammation is not static but exhibits time-dependent and disease context-dependent patterns, implying that the significance of a biomarker may vary across disease stages. Fourth, changes in peripheral inflammatory markers may not directly reflect brain-specific processes, necessitating careful interpretation of blood-based biomarkers. Finally, the field would greatly benefit from standardized reporting and validation of biofluid biomarkers to ensure reproducibility and facilitate their integration into the biological criteria for AD. Adopting this framework will enhance the rigor of biomarker research and its translation into clinical practice. Clinical trial designs must accommodate multi-target interventions by employing adaptive designs and composite endpoints to capture changes in cognition, function, and biomarkers (Schneider et al., 2014; Wang J. et al., 2016). Future research directions encompass the integration of artificial intelligence with multi-omics data to predict therapeutic responses, as well as the development of preventive immunomodulatory strategies targeting early-stage AD (Cong and Endo, 2022).

**TABLE 4:** Core neuroimmune-related biomarkers in Alzheimer’s Disease.

Biomarker	Biological Process	AD Relevance	Key Point
TSPO-PET	Microglial activation	Correlates with Aβ /tau and cognitive decline.	Pro: Non-invasive imaging. Con: Low specificity.
GFAP	Astrocyte reactivity	Strongly associated with Aβ pathology.	Pro: Minimally invasive, excellent biomarker. Con: May reflect systemic inflammation.
Plasma sTREM2	Microglial response	Elevated in AD, links to tau-induced pathogenesis.	Pro: Directly reflects TREM2 pathway activity.
CSF YKL-40	Neuroinflammation	Correlates with neurodegeneration rate.	Pro: CNS-specific. Con: Invasive sampling.

The interpretation of these biomarkers should adhere to a rigorous frame-work that acknowledges: (1) the insufficiency of a single marker to describe an entire biological cascade; (2) the need for simultaneous measurement of multiple markers; (3) the limitation of association studies in inferring mechanisms; (4) the dynamic, spatiotemporal patterns of neuroinflammation; (5) the potential disconnect between peripheral and central inflammation; (6) the necessity for standardized reporting. Adapted from principles outlined by Bettcher et al. (2025). TSPO-PET, Translocator Protein-Positron Emission Tomography; GFAP, Glial Fibrillary Acidic Protein; sTREM2, soluble Triggering Receptor Ex-pressed on Myeloid cells 2; CSF, Cerebrospinal Fluid; AD, Alzheimer’s Diseas-e; CNS, Central Nervous System; Aββ, Amyloid-beta.

## Discussion and Conclusion

This study systematically elucidates the paradigm shift in the pathological mechanisms of AD pathogenesis from the Aβ hypothesis to the neuroimmune network perspective. This paradigm transition underscores the increasingly recognized role of neuroimmune interactions and intercellular communication as integral components of the disease progression, which interacts with canonical Aβ and tau pathologies. Analytical findings indicate that microglial and astrocytic dysfunctions constitute critical drivers of AD pathogenesis. Upon transitioning to the disease-associated microglia (DAM) state, microglia exhibit diminished Aβ clearance capacity and release pro-inflammatory factors, exacerbating neuroinflammation. Concurrently, astrocytes lose their homeostatic support functions and acquire neurotoxic properties. These discoveries emphasize the crucial significance of disrupted brain immune homeostasis in AD pathogenesis.

Intercellular signaling molecules function as core mediators in pathological processes. Cytokine and chemokine networks sustain chronic inflammatory microenvironments. Extracellular vesicles (EVs) facilitate the propagation of Aβ and tau proteins. The complement system transitions from physiological synaptic pruning to pathological synaptic engulfment. These molecular mechanisms collectively contribute to neuronal damage and cognitive decline. Our research further reveals the involvement of peripheral immune cell infiltration and gut-brain axis dysregulation in expanding the pathological spectrum, thereby substantiating the multifactorial nature of AD.

Based on these mechanisms, therapeutic strategies are transitioning toward multi-target interventions. Immunomodulatory therapies (e.g., TREM2 agonists) can enhance the protective functions of microglia. Exosome-mediated drug delivery systems provide novel approaches for blood-brain barrier penetration. Combination therapies targeting both pathological proteins and neuroinflammation demonstrate synergistic effects. However, therapeutic development continues to face challenges such as disease heterogeneity and individual variability. Future endeavors should focus on developing personalized regimens and utilizing biomarkers for precise stratification.

Cutting-edge technologies such as spatial omics and single-cell sequencing have unveiled the spatiotemporal dynamics of cellular interactions. Human induced pluripotent stem cell-derived 3D organoid models offer more human-relevant research platforms. Emerging signaling pathways (e.g., cGAS-STING and ZBP1) have been identified as potential therapeutic targets. Systems biology approaches have facilitated the identification of key driver genes and network pharmacology strategies. These technological advancements provide powerful tools for decoding the complex interaction networks in AD.

In conclusion, this review highlights the significant and interconnected role of the neuroimmune network within the multifaceted landscape of AD pathology. Early interventions aimed at reshaping healthy intercellular communication may offer new hope for halting disease progression. Future research should focus on personalized therapies, multi-omics integration, and clinical trial optimization. Ultimately, multi-target strategies hold promise for improving clinical outcomes in AD.
